# Enhancing Patient Care: A Review of Physical Health Equipment in CMHTs

**DOI:** 10.1192/bjo.2025.10512

**Published:** 2025-06-20

**Authors:** Dilshana Nafisa Bapakunhi, Harini Bandela

**Affiliations:** Essex Partnership University NHS Foundation Trust, Colchester, United Kingdom

## Abstract

**Aims:** Individuals with mental illness face a higher risk for cardiovascular and metabolic disorders, exacerbated by psychotropic medications. Physical health assessments in CMHTs are crucial to prevent undiagnosed conditions and ensure proper care.

Guidelines emphasize the need for essential equipment for thorough assessments, as missing tools can hinder care and lead to misdiagnosis. This audit follows the POMH Valproate audit, which identified gaps in equipment availability in CMHTs across Essex.

The aim is to assess whether CMHTs have the necessary equipment for physical examinations according to trust policy, ensuring service quality by maintaining properly stocked and functional items.

**Methods:** This audit was conducted trust-wide across 10 CMHTs in North East, Mid, West, and South Essex from July to December 2023. A standardized proforma, aligned with the Physical Healthcare Trust policy, was used to assess equipment availability. Compliance was measured as the percentage of required items present and functional.

The audit followed these steps:

Initial Contact: We contacted the manager of each CMHT and liaised with assigned personnel responsible for physical health equipment.

Site Visits: We visited each centre, met with the physical health lead nurse (where available), and gathered data on equipment availability.

Equipment Assessment: We assessed all required equipment in collaboration with the nurse responsible for physical health and the examination room.

Discussion and Analysis: We discussed reasons for missing equipment and challenges in maintaining compliance.

**Results:** No site met the 100% compliance target. Key findings include:

Highest compliance: 76.6%.

Most CMHTs: 60-70% compliance.

Lowest compliance: 46.6%.

Commonly missing items: Pentorch, ophthalmoscope, otoscope, tongue depressors, reflex tendon hammer, tuning forks, peak flow meters.

Findings were presented to the Physical Health Sub-Committee and the Medicine Management Committee. Recommendations include appointing leads in each CMHT to oversee equipment checks and ensuring trust policy visibility in clinic rooms.

Following the audit, missing and non-functional equipment was restocked. Measures were taken to verify that all items were fully operational and accessible for healthcare professionals when needed. Physical examination rooms in CMHTs were also checked to ensure that the policy was visibly displayed and regularly reviewed for compliance.

**Conclusion:** The availability of essential physical health equipment is crucial for adhering to assessment guidelines. Gaps in equipment availability were identified, prompting corrective actions such as restocking missing items and appointing responsible leads. These steps aim to enhance patient care by ensuring thorough and effective physical health assessments.

